# The Role of 11-Oxygenated Androgens and Endocrine Disruptors in Androgen Excess Disorders in Women

**DOI:** 10.3390/ijms25179691

**Published:** 2024-09-07

**Authors:** Jana Vitku, Anezka Varausova, Tereza Skodova, Lucie Kolatorova, Michala Vosatkova, Josef Vcelak, Jana Vrbikova, Marketa Simkova, Michaela Svojtkova

**Affiliations:** 1 Department of Steroids and Proteofactors, Institute of Endocrinology, Narodni 8, 116 94 Prague, Czech Republic; avarausova@endo.cz (A.V.); tskodova@endo.cz (T.S.); lkolatorova@endo.cz (L.K.); msimkova@endo.cz (M.S.); msvojtkova@endo.cz (M.S.); 2 Department of Clinical Biochemistry, Institute of Endocrinology, Narodni 8, 116 94 Prague, Czech Republic; mvosatkova@endo.cz; 3 Department of Molecular Endocrinology, Institute of Endocrinology, Narodni 8, 116 94 Prague, Czech Republic; jvcelak@endo.cz; 4 Department of Clinical Endocrinology, Institute of Endocrinology, Narodni 8, 116 94 Prague, Czech Republic; jvrbikova@endo.cz

**Keywords:** polycystic ovary syndrome, idiopathic hirsutism, endocrine disruptor, bisphenol A, bisphenol S, 11-oxygenated androgen, 11-ketotestosterone, 11-hydroxytestosterone

## Abstract

Polycystic ovary syndrome (PCOS) and idiopathic hirsutism (IH) are androgen excess disorders requiring the determination of classic androgen levels for diagnosis. 11-oxygenated androgens have high androgenic potential, yet their clinical value in those disorders is not clear. Additionally, the role of endocrine disruptors (EDs), particularly in IH, remains understudied. We analyzed 25 steroids and 18 EDs in plasma samples from women with IH, PCOS, and controls using LC-MS/MS. Cytokine levels and metabolic parameters were assessed. Comparisons included non-obese women with PCOS (n = 10), women with IH (n = 12) and controls (n = 20), and non-obese versus obese women with PCOS (n = 9). Higher levels of 11-oxygenated androgens were observed in women with PCOS compared to those with IH, but not controls. Conversely, 11-oxygenated androgen levels were lower in women with IH compared to controls. Cytokine levels did not differ between women with IH and controls. Bisphenol A (BPA) levels were higher in obese women with PCOS compared to non-obese women with PCOS. Bisphenol S occurrence was higher in women with PCOS (90%) compared to controls (65%) and IH (50%). Significant correlations were found between androgens (11-ketotestosterone, androstenedione, testosterone) and insulin and HOMA-IR, as well as between immunomodulatory 7-oxygenated metabolites of DHEA and nine interleukins. Our data confirms that PCOS is a multiendocrine gland disorder. Higher BPA levels in obese women might exacerbate metabolic abnormalities. IH was not confirmed as an inflammatory state, and no differences in BPA levels suggest BPA does not play a role in IH pathogenesis.

## 1. Introduction

Androgen excess disorders, or hyperandrogenism, are common endocrinological conditions affecting around 5–20% of premenopausal women [1,2]. Manifestations of hyperandrogenic states include hirsutism, androgenic alopecia, ovulatory dysfunction, and even virilization and masculinization [3]. Androgen excess disorders have various identifiable causes including non-classic adrenal hyperplasia, hyperandrogenic insulin-resistant acanthosis nigricans syndrome, and androgen-secreting neoplasms. Alternatively, androgen excess disorders identified by exclusion, such as idiopathic hirsutism (IH) and polycystic ovary syndrome (PCOS), are termed disorders of functional androgen excess [3]. PCOS accounts for the majority of hyperandrogenic states (72–82%), while patients with IH account for 4.7–7.6% [3,4]. 

For an exact diagnosis of androgen excess disorders, determining classic androgens (androstenedione—A4, testosterone—T, dehydroepiandrosterone—DHEA and its sulfate—DHEAS), together with additional analytes (17-hydroxyprogesterone and sex hormone-binding globulin—SHBG), is crucial. In recent years, the 11-oxygenated derivatives of classic androgens (A4, T, dihydrotestosterone—DHT) have been gaining attention because of their non-negligible or even high androgenic potential, which is sometimes comparable to the potency of classic androgens [5]. They are derived from 11β-hydroxyandrostendione (11βOHA4) of adrenal origin, which serves as a precursor for the more efficient 11-oxygenated androgens (11β-hydroxytestosterone—11OHT and 11-ketotestosterone—11KT, 11β-hydroxydihydrotestosterone and 11-ketodihydrotestosterone).

The determination of these androgens could be especially helpful in diagnosing hyperandrogenic disorders. In fact, their significance has already been studied in PCOS. Although PCOS is primarily an ovarian disorder, the function of the adrenal glands is also disturbed. One study found significantly higher levels of 11-oxygenated androgens (11βOHA4, 11-ketoandrostenedione—11KA4, 11OHT and 11KT) together with higher levels of classic androgens in women with PCOS compared to controls [6]. Another group reported higher levels of either classic androgens, 11-oxygenated androgens, or both in women with PCOS [7], suggesting that the profile of circulating androgens could vary among distinct subgroups of women with PCOS. 

PCOS is characterized by metabolic disturbances as well as low-grade inflammation [8,9,10,11]. Changes in cytokine composition [12], higher C-reactive protein levels [13], and white blood cells counts [9,14,15] in women with PCOS are indicative of a disturbed immune system. One possible connection between the immune and endocrine systems could be the 7-oxygenated metabolites of DHEA. These metabolites, especially 7α-hydroxydehydroepiandrosterone (7αOHDHEA), have immunomodulatory [16,17,18], anti-glucocorticoid [19] and antioxidative effects [20]. No studies have yet dealt with possible inflammation in IH. Thus, the determination of 7-oxygenated metabolites of DHEA together with cytokine levels in androgen excess disorders could be of interest.

The underlying cause of IH is thought to be a primary increase in the activity of 5α-reductase in the skin, likely involving both isoenzyme types along with a potential change in androgen receptor function and/or increased ovarian thecal activity stimulated by hyperinsulinemia [2,21]. Although in PCOS, hyperandrogenism has established consequences including glucose intolerance, hypertension, and obesity [2], studies investigating metabolic outcomes in women with IH have been limited by small sample sizes or inconsistent design and are also conflicting [22,23]. IH is also understudied from the immune system point of view compared to PCOS. Because of the high androgenic potential of 11-oxygenated androgens, we hypothesize that their levels, which are not routinely assessed, could explain the discrepancy between the intensity of clinical signs of hyperandrogenism and normal plasma T and DHT levels. No studies have yet focused on the role of 11-oxygenated androgens in IH, although there are some studies that have evaluated 11-oxygenated androgens and self-assessed hirsutism or hirsutism scores in women with PCOS [24,25] or without [26], but with mixed results.

In addition to the endogenous steroids that are involved in the pathogenesis of androgen excess disorders, the impact of environmental factors including exogenous substances should also be taken into account. Endocrine disruptors (EDs) are natural (e.g., phytoestrogens) or man-made chemicals that can interfere with the endocrine system, interacting with hormone receptors, influencing gene expression and the activity of enzymes involved in hormone biosynthesis, or acting through epigenetic mechanisms [27]. EDs are present in numerous everyday products: (1) in personal-care products as antimicrobial agents (parabens, triclosan) or UV-filters (oxybenzone); (2) in plastic and polymer products as plasticizers (phthalates) or used for food and drink packaging (bisphenols, phthalates, perfluoroalkylated substances, nonylphenol); (3) in household and industrial products as surfactants in detergents (alkylphenols), stain- and water-resistant coatings for fabrics and carpets (perfluoroalkylated substances) or as flame retardants (polybrominated diphenyl ethers; chlorinated, brominated, or organophosphate flame retardants); (4) as agricultural chemicals (pesticides, herbicides, fungicides). Their impact on human health and disease has been the subject of much recent research, revealing associations with a range of conditions including reproductive issues (e.g., [28,29], developmental disorders [30], metabolic disturbances [31], and some types of cancers [28,32]. Some EDs can have obesogenic effects that can result in disturbances in energy homeostasis [33,34].

Among androgen excess disorders, PCOS has been the most well studied in the context of possible influence by EDs. Higher levels of bisphenol A (BPA) in women with PCOS have already been reported by multiple groups including ours (e.g., [12,35,36,37]), as reviewed in Urbanetz et al. [38]. The roles of other less well-known EDs have been less explored. Serum bisphenol S (BPS) was reported to be higher in PCOS women compared to controls, while the levels of BPA did not differ between these groups [39]. To the best of our knowledge, no study has yet dealt with the role of EDs in IH.

Because of the close interconnections among endocrine, immune, and metabolic processes, in this study, we aimed to take a complex picture of women with PCOS and women with IH in terms of four panels of analytes:

(1) metabolic parameters (parameters of insulin resistance, liver enzymes, lipids and glucose concentrations)
(2) unconjugated and conjugated levels of 25 steroid hormones, with emphasis on classic androgens, 11-oxygenated androgens, and 7-oxygenated metabolites of DHEA
(3) unconjugated levels of 18 EDs including bisphenols (bisphenol A-BPA, BPS, BPF, BPAF, BPAP, BPZ, BPP), parabens (methyl-, ethyl-, propyl-, butyl-, benzyl- paraben), benzophenones (benzophenon-3 = oxybenzone, benzophenone-1), phytoestrogens (daidzein, genistein, S-equol), and nonylphenol (NP)
(4) concentrations of 27 cytokines

More specifically, we wanted to answer the following questions:

(1) Do levels of 11-oxygenated androgens differ between age- and BMI-matched women with PCOS (n = 10), women with IH (n = 12), and the control group (n = 20)? Do they contribute to the circulating androgen pool in these women?
(2) Do levels of EDs differ between women with PCOS, women with IH, and the control group? Can EDs be involved in the pathogenesis of these disorders?
(3) Is IH also a low-grade inflammation state similar to PCOS?
(4) Are there differences in the levels of 11-oxygenated androgens, EDs, cytokines, and immunomodulatory 7-oxygenated metabolites of DHEA between normal-weight (n = 10) and obese (n = 9) women with PCOS? Is there an effect of obesity?
(5) What are the relationships among 11-oxygenated androgens, EDs, metabolic parameters, and cytokine levels?

The analysis of a broad spectrum of analyte panels allowed us to study the complex relationships among the endocrine, immune, and metabolic processes in androgen excess disorders in women, helping to fill the gap in research focusing on non-classic androgens and endocrine disruptors (EDs) in these disorders.

## 2. Results

### 2.1. Basic and Metabolic Characteristics

The basic and metabolic characteristics of each group are presented in Table 1. None of the characteristics were significantly different from other groups as assessed by the Kruskal–Wallis test, except for hemoglobin A1c and alanin aminotransferase (ALT), which were higher in women with PCOS and women with IH compared to the control group. However, both hemoglobin A1c and ALT levels in all groups were within the normal reference ranges of our laboratory, which is 20–42 mmol/mol for hemoglobin A1c and 0.17–0.58 µkat/L for ALT.

### 2.2. Cytokine Profiles in Women with PCOS, Women with IH, and Correlation Analysis

The levels of cytokines and their comparison among BMI-matched groups can be found in Table S1. Cytokine levels did not differ between women with IH, women with PCOS and healthy controls with one exception; platelet-derived growth factor (PDGF-BB) levels were higher in women with PCOS in comparison with controls (*p* < 0.01). An isolated elevation of one cytokine probably has no relevance.

**Figure 1:**
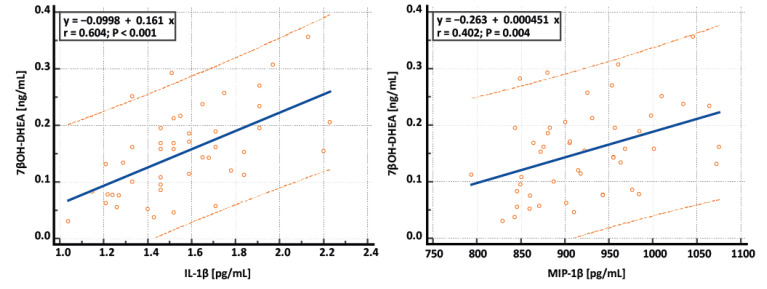
Representative scatterplots showing relationships between 7β-hydroxydehydroepiandrosterone (7βOH-DHEA) and interleukin 1β (IL-1β) and 7βOH-DHEA and macrophage inflammatory protein 1β (MIP-1β). Blue lines represent regression lines, and orange dotted lines represent 95% prediction intervals.

The measured cytokines were further correlated with levels of 7-oxygenated metabolites of DHEA, which possess immunomodulatory properties. For the correlation analysis, all premenopausal women were included (12 women with IH, 20 controls, and 10 non-obese and 9 obese women with PCOS). Spearman’s correlations are summarized in Table 2, and representative scatterplots are depicted in Figure 1. Positive correlations can be seen between 7-oxygenated metabolites of DHEA and six pro-inflammatory cytokines (interleukins IL-1β, IL-2, IL-12, IL-17, macrophage inflammatory protein 1α—MIP1α, MIP1β) as well as two anti-inflammatory cytokines (IL-1ra, IL-13), indicating a mutual connection between the endocrine and immune systems through immunomodulatory steroid hormones and interleukins.

### 2.3. Hormonal Profiles of Women with IH and Women with PCOS

**Figure 2:**
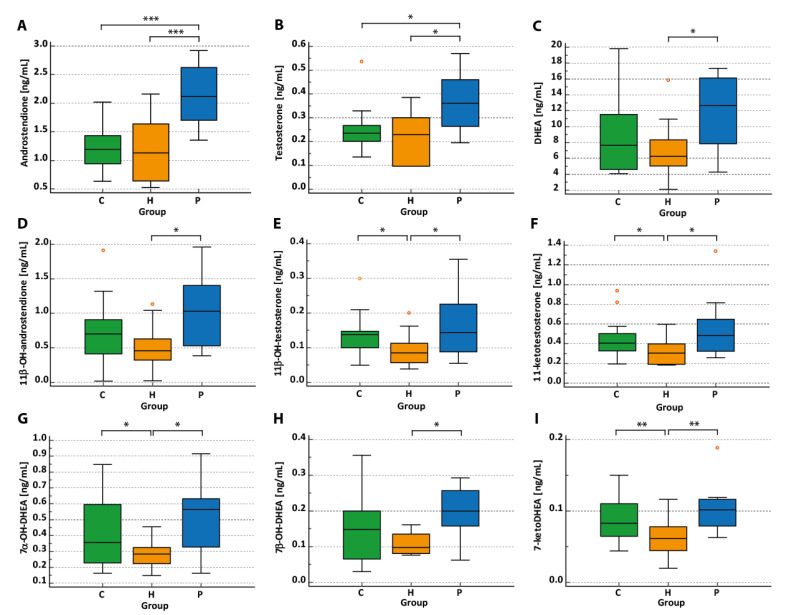
Plasma concentrations of classic androgens (**A**–**C**), 11-oxygenated androgens (**D**–**F**), and 7-oxygenated androgens (**G**–**I**) in BMI- and age-matched healthy subjects (Group C, n = 20), in women with idiopathic hirsutism (Group H, n = 12) and women with PCOS (Group P, n = 10). * Significant at *p* < 0.05. ** Significant at *p* < 0.01. *** Significant at *p* < 0.001. Red circles indicate outliers.

Hormone levels in each group and multiple comparisons between BMI-matched groups are shown in Table 3, with both unconjugated (Table 3) as well as conjugated fractions (Table S2) being analyzed. Box-Whisker plots for selected androgens in the three groups are depicted in Figure 2. As expected, the levels of classic androgens were higher in women with PCOS compared to controls (and women with IH). 11-oxygenated androgens were significantly higher in women with PCOS compared to women with IH and non-significantly compared to the control group. Similarly, when we compared all women with PCOS (n = 19) with women with IH, 11-oxygenated androgens (11OHT, 11KT) were significantly higher in patients with PCOS (*p* < 0.05 for both androgens).

Put differently, 11-oxygenated androgens, specifically 11OHT and 11KT, were lower in women with IH compared to controls. Interestingly, these findings suggest that women with IH have a distinct profile of circulating androgens. Similar results were found for the 7-oxygenated metabolites of DHEA: 7αOHDHEA and 7KDHEA were significantly lower in women with IH compared to controls.

When comparing T and 11KT levels, 11KT levels were significantly higher than T levels in our study across all groups (*p* < 0.0001 for the PCOS group, and *p* < 0.01 for the control group and the group of women with IH).

The levels of conjugated steroids (Table S2) did not differ among the groups except for 11-deoxycorticosterone (11-DOC), which was higher in women with PCOS compared to controls and women with IH, and conjugated 7KDHEA, which was significantly lower in women with IH compared to controls (*p* < 0.01), similarly as for its unconjugated form.

### 2.4. Correlations of Classic and 11-Oxygenated Androgens with Metabolic Parameters

Spearman’s correlations were used to evaluate associations between classic and 11-oxygenated androgens on the one hand and metabolic parameters on the other (Table 4). Significant positive associations were found for 11KT, A4, and T with insulin and HOMA-IR, indicating the relation of these androgens to insulin resistance. Furthermore, A4 was negatively correlated with age and positively with ALT, DHT was negatively correlated with LDL cholesterol, and T positively with glucose as well as liver enzymes (ALT, aspartate transaminase—AST, gamma-glutamyl transferase—GGT).

### 2.5. ED Levels in Women with Idiopathic Hirsutism and PCOS and Their Correlations with Androgens, 11-Oxygenated Androgens, Metabolic Parameters and Immune Parameters

Our recently validated methods allow for the detection of 18 unconjugated—bioavailable—EDs. The most commonly detected EDs were BPA and BPS, followed by methylparaben (MP) and oxybenzone, with concentrations and occurrences given in Table 5. None of the levels of any EDs differed between the BMI- and age-matched groups.

Among the further analyzed EDs:

- Ethylparaben (EP) was detected in 15% of controls, 33% of women with IH, and 10% of women with PCOS.
- Propylparaben (PP) was detected in 10% of controls, 33% of women with IH, and 10% of women with PCOS.
- Benzophenone-1 was detected in 5% of controls, 17% of women with IH, and 10% of women with PCOS.
- Daidzein was detected in 40% of controls, 42% of women with IH, and 20% of women with PCOS.
- Genistein occurred in 15% of controls, 8% of women with IH, and 20% of women with PCOS.
- *S*-equol was detected in one sample of a woman with PCOS.
- Butylparaben (BP) was detected in one sample of a woman with IH.

BPF, BPZ, BPAF, BPAP, BPP, benzylparaben (BenzylP), and nonylphenol (NP) were not detected in any sample.

Correlations between EDs with the highest occurrences (BPA, BPS, MP and oxybenzone) and metabolic parameters are reported in Table S3. A significant negative correlation was found between BPS and ALT (*p* < 0.05), and there were nearly significant positive correlations of BPA with BMI and GGT (*p* = 0.056 and *p* = 0.054, respectively).

Correlations between EDs and the cytokine panel together with immunomodulatory 7-oxygenated metabolites of DHEA are given in Table S4. A significant negative correlation was found between IP-10 and BPS (r = −0.377, *p* < 0.01). Furthermore, BPS positively correlated with 7αOHDHEA and 7KDHEA (r = 0.292, *p* < 0.05 and r = 0.443, *p* = 0.001, respectively), suggesting the influence of the immune system through immunomodulatory steroids.

To complete the picture, Table S5 reports correlations between EDs with the highest occurrences (BPA, BPS, MP and oxybenzone) and classic as well as 11-oxygenated androgens. Of the evaluated analytes, BPA negatively correlated with A4 and T (*p* < 0.05 in both androgens).

### 2.6. Differences in Steroid, ED and Cytokine Levels between Normal-Weight and Obese Women with PCOS

The levels of certain cytokines differed in normal-weight women with PCOS compared to obese women with PCOS. Here we confirmed our results from a previous study [12]. where these groups were also compared; however, the study groups were slightly different. The levels of IL-1ra, IL-7, IL-13, and IFNγ were significantly higher in the group of obese women with PCOS in this study, confirming changes in the immune systems of this group. A comparison of the concentrations of all analyzed cytokines is shown in Table S6.

Comparisons of ED levels, AMH, and androgens between normal weight and obese women with PCOS are shown in Table 6. BPA levels in obese women with PCOS were significantly higher than in normal weight women with PCOS. Concentrations of AMH were higher in normal weight women with PCOS. No other differences between EDs and classic or 11-oxygenated androgens were found.

## 3. Discussion

In this study, we attempted to assess the roles of endogenous steroid hormones with an emphasis on classic and 11-oxygenated androgens, EDs that can interfere with the steroid hormone action, and the immune system represented by cytokine levels in women with IH and women with PCOS.

As expected, classic androgens were significantly higher in women with PCOS compared to controls. 11-oxygenated androgens were significantly higher in women with PCOS in comparison with women with IH, but non-significantly compared to the control group. This difference was significant in larger studies by O’Reilly et al., where the serum levels of 11βOHA4, 11KA4, 11βOHT, and 11KT were higher in 114 women with PCOS compared to the control group (n = 49) [6], by Tosi et al. where 11KT and 11OHT were higher in women with PCOS [24], and also by Taylor et al., where the levels of 11βOHA4 and 11OHT were higher in women with PCOS than non-PCOS controls [25]. In another study, the role of classic and 11-oxygenated androgens was evaluated in the light of two features of PCOS—polycystic ovary morphology and menstrual cycle prolongation. Classic androgens contributed more than 11-oxygenated, while the most significant contributor was AMH [40]. Our results were in line with this later study, with the levels of classic androgens as well as AMH significantly higher in PCOS women vs. controls. Furthermore, AMH levels were also different between non-obese and obese women with PCOS, with higher levels of AMH in the non-obese group. These results support those from larger multicenter cohorts where higher BMI has been associated with reductions in AMH [41, 42].

Similar to that of other authors [6,43,44], the most abundant 11-oxygenated androgen in our study was 11βOHA4 followed by 11 KT and 11OHT. In contrast to other studies [45,46], however, the levels of 11-oxygenated androgens were not associated with either age or BMI in our study. The reason might lie in the design of our study, where all groups were of similar ages and BMIs, except the group of obese women with PCOS. However, 11KT, along with T and A4, showed a strong correlation with insulin and HOMA-IR in our study. This supports the role of insulin in regulating AKR1C3 expression, leading to hyperandrogenemia, which in turn results in hyperinsulinemia and insulin resistance, creating a vicious cycle as discussed in the review by Storbeck and O’Reilly [47].

Surprisingly, in contrast to our hypothesis about possible higher amounts of 11-oxygenated androgens in women with IH, the levels were in fact lower. Therefore, the original assumption that the pathogenesis of IH can be explained by increased peripheral 5α-reductase enzyme activity and/or abnormalities of androgen receptor gene polymorphisms still remains [3,48]. We can further hypothesize that a rather lower circulating androgen pool (classic + 11-oxygenated androgens) might be compensated for by over-sensitization at the receptor level.

The levels of ALT and hemoglobin A1c were significantly higher in women with IH (as well as in women with PCOS) compared to the control group. Although still within reference ranges, these results suggest some influence of metabolism similarly as in women with PCOS. In one large prospective study, no changes in metabolic risk factors in women with IH compared to heathy controls were found [23]. On the other hand, in a metanalysis from the same research group, there were higher levels of fasting glucose and insulin in women with IH compared to the control group [22]. So there still remains a question if metabolic parameters should be evaluated in women with IH.

7-oxygenated metabolites of DHEA (7αOHDHEA and 7KDHEA) were found to be significantly lower in women with IH compared to controls. One reason may be the reduction, although not significant, of its precursor—DHEA. The immunomodulatory effects of 7-oxygenated DHEA metabolites can be seen in Table 5 expressed as Spearman’s correlations with the cytokine panel. 7-oxygenated metabolites of DHEA were positively correlated with eight cytokines, of which six are reported to be pro-inflammatory: IL-1β [49,50], IL-2 [51], IL-12 [52], IL-17 [53], MIP1α [54], MIP1β [55] and two anti-inflammatory IL-1ra [56], IL-13 [57]. These findings support the results of immunomodulatory properties of 7-oxygenated DHEA metabolites and suggest a further connection between the endocrine and immune systems apart from sex hormones and stress-related hormones [58,59].

There were no differences between any of the groups in levels of cytokines with one exception; higher levels of PDGF-BB, which is a stimulator of cell proliferation [60], were found in PCOS women compared to controls. The same observation was reported by another study [61]. Significantly different levels of some further cytokines were found in obese PCOS women compared to those with normal weight, similarly as in our previous study [12]. Further changes in cytokine composition in obese women with PCOS suggests that obesity significantly contributes to an inflammatory state in such women.

The association of BPA with PCOS has been extensively studied, showing a significant relationship (independent of obesity) [35,38,62,63]. In our study, BPA levels were significantly higher only in obese women with PCOS compared to normal-weight women with PCOS. Additionally, BPA correlated with BMI, although the correlation was only borderline significant (*p* = 0.056). Given BPA’s known obesogenic effects, which can stimulate adipogenesis through multiple mechanisms [34,64,65], its elevated levels in obese women with PCOS may further exacerbate metabolic abnormalities. These findings underscore the complexity of the interplay between endocrine disruptors like BPA and metabolic conditions, highlighting the need for further research to unravel the specific mechanisms through which BPA influences PCOS pathophysiology and obesity.

In addition to BPA, high levels of BPS were observed in women with PCOS and with IH, but the differences in concentrations were not statistically significant, contrary to findings by Jurewicz et al. [39] who reported higher serum BPS in women with PCOS and Zhan et al. who found BPS (and BPA, BPAF, and BPZ) to be positively associated with PCOS [66]. Interestingly, this association was stronger in women who were overweight or obese.

In our study, BPS negatively correlated with ALT levels, which would suggest a rather positive effect on liver enzymes. In contrast, an experimental study involving the administration of BPS to Sprague-Dawley rats at doses of 30, 60, and 120 mg/kg BW/day for 30 days reported an increase in ALT levels [67]. Clearly, the findings from a rat study cannot be directly extrapolated to humans, particularly since the doses administered in the rat study were significantly higher than the environmental exposures experienced by women in our study. Additionally, humans are exposed to a complex mixture of EDs, which can interact with each other, complicating the attribution of effects to individual substances.

This study has some limitations. First and the most relevant is the small number of cases included in our study, since a small sample size makes only statistically significant large effects detectable, and small effects can be missed. Second, our method does not include some of the 11-oxygenated androgens. Our future studies will be focused on the validation of further 11-oxygenated androgens (11KA4, 11OHDHT and 11KDHT) and their incorporation into the method to provide a complex picture about non-classic androgens. A strength of our study lies in the detailed independent evaluations of patients’ clinical states by two experts on steroid diagnostics, resulting in well characterized groups. The experts agreed on the diagnosis in 100% of cases. Furthermore, measurements of EDs and steroids were performed using published LC-MS/MS methods validated according to FDA guidelines, possessing high sensitivity [68,69]. Additional analytes, such as biochemical markers, LH, FSH, AMH, and cytokines, were determined using established methods in our laboratory, requiring no further optimization..

## 4. Materials and Methods

### 4.1. Study Population

The women with IH and PCOS and the healthy women were recruited by the Institute of Endocrinology, Prague, Czech Republic. This study was approved by the Ethical Committee of the Institute of Endocrinology under a protocol nr.: 10.12.12.2016, and the trial was conducted in accordance with the Declaration of Helsinki. All participants signed an informed consent before entering this study. All PCOS patients met the Rotterdam European Society of Human Reproduction and Embryology criteria (2004) and National Institute of Health criteria (1990).

Primarily, 76 women with suspected PCOS or hirsutism were examined. From this group, women with PCOS and women with idiopathic hirsutism (IH) were differentiated. Only women whose diagnosis was confirmed by two independent clinicians with expertise in steroid diagnostics were included in the groups. Recruitment took place over a period of 10 months.

The group of women with IH (n = 12) were diagnosed by hirsutism, normal classic androgen levels, and no ovulatory dysfunction after the exclusion of PCOS, non-classic adrenal hyperplasia, hyperandrogenic insulin-resistant acanthosis nigricans syndrome, and androgen secreting tumors. Further exclusion criteria for both groups included other oncologic or autoimmune diseases, nutritional disorders, and excessive alcohol consumption. Additionally, none of the women could be receiving treatments that could affect steroid hormone metabolism, such as corticosteroids (including topical or inhaled forms), hormonal contraception, insulin sensitizers, lipid-lowering drugs, antidepressants, antiepileptics, neuroleptics, or antihypertensives.

This study focused on the examination of female patients. Afterward, they received standard treatment and monitoring for PCOS or hirsutism, in accordance with the guidelines of the Androgen Excess and PCOS Society. Treatment options were discussed and explained, and the most suitable one was chosen in consultation with the patient, based on her preferences.

Women included in this study as healthy controls showed no signs of high androgen levels and had a regular menstrual cycle. All included women were of European descent.

### 4.2. Materials and Chemicals

Reference standards of steroid hormones, Eds, and internal standards were purchased from Koch-Light Laboratories Ltd. (Suffolk, UK), Merck (Darmstadt, Germany), Chromservis (Prague, Czech Republic), Steraloids (Newport, RI, USA), Cambridge Isotope Laboratories, Inc. (Tewksbury, MA, USA), Chiron (Trondheim, Norway), EQ Laboratories GmbH (Augsburg, Germany), Cayman Chemical Company (Ann Harbor, MI, USA), or Toronto Research Chemicals Canada (Toronto, ON, Canada) as reported in recent papers [68,69,70]. Trimethylchlorsilane (TMCS) was from Merck (Darmstadt, Germany). Formic acid (FA, eluent additive for LC-MS, ≥96%) and ammonium fluoride (NH4F, eluent additive for LC–MS, ≥98.0%) were obtained from VWR International (Stribrna Skalice, Czech Republic). Methanol (≥99.9%,) and water were from Honeywell Research Chemicals (Charlotte, NC, USA). Ethyl acetate (≥99.9%) and n-hexane (≥99%) were purchased at VWR International (Wayne, PA, USA). All solvents and reagents were of LCMS grade.

### 4.3. Sample Collection

Blood samples were collected between 7 and 9 am taking into account the circadian rhythm of the steroid hormones [71]. All women were at the follicular phase of their menstrual cycle—i.e., between day 1 and day 5 of the cycle. All steps in the sample collection and protocol were controlled for bisphenol and paraben contamination [72,73]. Plasma samples were stored at −20 °C until analysis.

### 4.4. Analysis of Biochemical Markers, Hormone and Cytokine Levels

After standardized blood collection into BD Vacutainer^®^ blood collection tubes (Becton, Dickinson and Company, Franklin Lakes, NJ, USA), the blood was separated by 10-min centrifugation (1450 RCF, 18 °C). Plasma levels of the biochemical markers were subsequently analyzed using routine methods on a Cobas^®^ 6000 analyzer (Roche, Mannheim, Germany) from approximately 300 µL of plasma. The biochemical markers include the following: albumin, LDL and HDL cholesterol, cholesterol, triglycerides, hemoglobin A1c, liver enzymes [alkaline phosphatase (ALP), alanine transaminase (ALT), aspartate transaminase (AST), gamma-glutamyl transferase (GGT)], glucose, and insulin. HOMA-IR (Homeostatic Model Assessment for Insulin Resistance) was calculated from glucose and insulin levels according to the following formula: fasting insulin (µIU/mL) × fasting glucose (mmol/L)/22.5 [74].

Anti-Müllerian hormone (AMH), luteinizing hormone (LH), and follicle stimulating hormone (FSH) were measured using an electro-chemiluminescence immunoassay on a Cobas^®^ 6000 analyzer (Roche, Mannheim, Germany) with routine methods from a 300 µL plasma sample. The levels of sex hormone binding globulin (SHBG) were measured using an immunoradiometric assay (Immunotech, Marseille, France) on a Stratec automatic analyzer (Birkenfeld, Germany) from a 50 µL sample.

A Bio-Plex Pro™ Human Cytokine 27-plex assay kit from Bio-Rad Laboratories, Inc. (Hercules, CA, USA) was used for the determination of 27 cytokines [(fibroblast growth factor basic (FGF basic), eotaxin, granulocyte colony stimulating factor (G-CSF), granulocyte macrophage colony stimulating factor (GM-CSF), interferon γ (IFN-γ), interleukins—IL-1β, IL-1ra, IL-2, IL-4, IL-5, IL-6, IL-7, IL-8, IL-9, IL-10, IL-12 (p70), IL-13, IL-15, and IL-17A—interferon γ-inducible protein, 10 kDa (IP-10), monocyte chemotactic and activating factor (MCAF/MCP-1), macrophage inflammatory protein 1α (MIP-1α), MIP-1β, platelet-derived growth factor, two B subunits (PDGF-BB), chemokine regulated on activation, normal T expressed and secreted (RANTES), tumor necrosis factor α (TNF-α), and vascular endothelial growth factor (VEGF)]. A volume of 50 µL of 4× diluted plasma was used for the analysis. Samples were thawed immediately before analysis and centrifuged at 1000 RCF (4 °C, 15 min) to remove particulates from the samples. All subsequent preparation steps were performed on ice and followed the manufacturer’s protocol. The analysis was run on a Bio-Plex^®^ 200 Luminex analyzer (Bio-Rad Laboratories, Inc., Hercules, CA, USA).

### 4.5. Analysis of Steroid Hormones and Endocrine Disruptors (EDs)

Steroid hormones and EDs were measured according to previously published methods [68,69,70,75]. Unconjugated and conjugated fractions of steroids and the unconjugated fraction of EDs were assessed. The following steroids were measured: C_18_ steroids—estrone (E1), 17β-estradiol (E2), estriol (E3); C_19_ steroids—dehydroepiandrosterone (DHEA), androstenedione (A4), testosterone (T), epitestosterone (EpiT), dihydrotestosterone (DHT), 11β-hydroxyandrostendione (11βOHA4), 11-hydroxytestosterone (11OHT), 11-ketotestosteone (11KT), 7α-hydroxydehydroepiandrosterone (7αOHDHEA), 7β-hydroxydehydroepiandrosterone (7βOHDHEA), and 7-ketodehydroepiandrosterone (7KDHEA); C_21_ steroids—progesterone, pregnenolone, 17-hydroxypregnenolone, 17-hydroxyprogesterone, cortisol, cortisone, 21-deoxycortisol, 11-deoxycortisol, 11-deoxycorticosterone, corticosterone, and aldosterone.

The measured EDs included bisphenols (bisphenol A-BPA, BPS, BPF, BPAF, BPZ, BPAP, BPP), parabens (methylparaben-MP, ethylparaben-EP, propylparaben-PP, butylparaben-BP, benzylparaben-benzylP), benzophenones (benzophenone 1, benzophenone 3 = oxybenzone), nonylphenol, and phytoestrogens (daidzein, genistein, *S*-equol).

#### 4.5.1. Sample Preparation

The preparation of the samples for ED and steroid analyses were the same [68,69]. Briefly, 10 μL of an internal standard (IS) mixture was added to 500 μL of a plasma sample and diluted with 500 μL of 0.9% saline. Liquid-liquid extraction (LLE) was performed using a hexane ethylacetate mixture (3:2, *v*/*v*). The organic phase was used to determine unconjugated steroids and EDs, while the aqueous phase was used to determine the conjugated steroids. The organic phase was evaporated to dryness using a vacuum concentrator and subsequently diluted with 100 μL of 50% methanol. Then, the samples were purified by centrifugation (4650× *g*, 5 min), and 90 μL was transferred to the vial with a glass insert. The aqueous phase samples were precipitated by 1.5 mL of iced methanol and centrifugated (4650× *g*, 10 min). The supernatant was transferred into a clean glass tube and again vacuum evaporated to dryness. Deconjugation was performed by adding 500 µL of 1 M trimethylchlorosilane (TMCS) to each sample, followed by incubation for 1 h at 65 °C. After incubation, 100 mg of sodium bicarbonate was added, and the mixture was evaporated to dryness. The residues were reconstituted in 100 μL of 50% methanol and processed similarly to the organic phase (LLE and centrifugation). An injection volume of 20 μL was used for the determination of steroid hormones (excluding estrogens and aldosterone) while 30 μL was used for the determination of EDs (and estrogens and aldosterone).

#### 4.5.2. LC-MS/MS Parameters

The analysis was performed on an ExionLC AD liquid chromatography system (Sciex, Concord, ON, Canada) coupled with a Sciex QTRAP 6500+ mass spectrometer (Sciex, Concord, ON, Canada). For the measurement of steroid hormones (except estrogens and aldosterone), the mass spectrometer operated in positive electrospray ionization (ESI) mode. LC was performed with a Kinetex C18 column (100 mm × 3.0 mm, 2.6 μm) and a Security Guard ULTRA cartridge system (UHPLC C18 for 3 mm ID column), both obtained from Phenomenex (Torrance, CA, USA). Water with 0.1% formic acid (FA) and methanol with 0.1% FA were used as mobile phases. Detailed LC-MS/MS conditions are given in Simkova et al. [68]. For the measurement of EDs, estrogens, and aldosterone, the mass spectrometer operated in the negative ESI mode. For separation, a Kinetex Biphenyl column (100 mm × 3 mm, 2.6 μm; Phenomenex, Torrance, CA, USA) and the corresponding Security Guard ULTRA cartridge system (UHPLC C18 for 3 mm ID biphenyl column; Phenomenex, Torrance, CA, USA) were used. Water and methanol were employed as mobile phases, with 6 mmol of ammonium fluoride infused post-column in the water to improve sensitivity. Detailed parameters are published elsewhere [69].

### 4.6. Statistical Analysis

Data below LLOQ were replaced by LLOQ/√2 according to Hornung et al. [76]. Due to the non-Gaussian distribution of the data, a log transformation was applied. When the transformation was effective, ANOVA followed by Scheffé’s multiple comparison test was used. When the log transformation was not effective [(Shapiro–Wilk test rejected normality or Levene’s test for equality of variances was positive (*p* < 0.05)], the Kruskal–Wallis test with untransformed data followed by the Conover–Iman post hoc test was used for comparisons of the three groups (controls, women with IH, and women with PCOS). While the majority of the data were of non-Gaussian distribution, Spearman’s correlations were used for all correlation analyses. MedCalc^®^ Statistical Software version 22.019 (MedCalc Software Ltd., Ostend, Belgium) was used for statistical testing.

## 5. Conclusions

From the correlation analysis, 7-oxygenated metabolites of DHEA were associated with six pro-inflammatory and two anti-inflammatory cytokines, confirming the immunomodulatory properties of these steroid hormones. Furthermore, significant positive correlations were observed between androgens (11KT, T, A4) and insulin and HOMA-IR, as well as between T and glucose and liver enzymes (ALT, AST, GGT). These findings suggest an interplay between androgen levels and metabolic function, highlighting the influence of these hormones on insulin resistance and metabolic health.

Our data confirms that PCOS is a multi-endocrine gland disorder, affecting both the gonads and the adrenals, with 11-oxygenated androgens contributing to the circulating pool, while 11KT levels were significantly higher than T levels in all groups (*p* < 0.0001 for PCOS group, and *p* < 0.01 for control group and group of women with IH). Low-grade inflammation was primarily observed in obese women with PCOS, reflecting the adverse effects of obesity. Additionally, BPA levels were associated with obesity, and higher levels found in obese women with PCOS may further exacerbate their metabolic abnormalities.

In contrast, idiopathic hirsutism appears to affect the adrenals differently than PCOS, with lower 11-oxygenated androgens of adrenal origin. Along with lower levels of DHT and normal levels of other classic androgens, we can speculate that the clinical signs of androgen excess in IH are caused by an oversensitivity of androgen receptors as a compensatory mechanism for the lower circulating androgenic pool. Certain changes in metabolic parameters were also observed. Finally, IH was not confirmed as an inflammatory state based on cytokine levels in our study, and although the occurrence of BPA was high in women with IH (92%), the levels were not significantly different from controls, suggesting that BPA does not play a role in the pathogenesis of IH.

## Figures and Tables

**Table 1:** Basic and metabolic characteristics of the control group, the group of women with IH and the group of women with PCOS.

	Controls (n = 20)	Women with IH (n = 12)	Women with PCOS (n = 10)	*p*-Value	Multiple Comparison
Age [y]	29 (26–38)	33 (27–37)	28 (24–32)	0.434	
BMI [kg/m^2^]	20.4 (19.9–23.2)	23.7 (20.3–25.3)	21.6 (18.5–24.5)	0.328	
HOMA-IR	1.3 (0.9–1.8)	1.7 (1.2–2.2)	1.7 (1.3–2.7)	0.125	
Insulin [mIU/L]	5.9 (3.9–7.7)	6.9 (5.2–10.0)	8.1 (6–11.6)	0.15	
Glucose [mmol/L]	5.0 (4.9–5.3)	5.2 (5.0–5.4)	5.2 (4.9–5.3)	0.539	
Hemoglobin A1c [mmol/mol]	32.0 (31.0–33.0)	35.5 (32.0–38.0)	34 (34–35)	**<0.001**	C<H, P
Albumin [g/L]	47 (45–50)	48 (46–50)	50 (47–52)	0.226	
ALP [µkat/L]	0.88 (0.82–1.00)	0.94 (0.82–1.14)	1.22 (0.94–1.48)	0.129	
AST [µkat/L]	0.32 (0.30–0.37)	0.34 (0.31–0.36)	0.33 (0.30–0.46)	0.724	
GGT [µkat/L]	0.22 (0.18–0.27)	0.22 (0.20–0.26)	0.27 (0.21–0.36)	0.239	
ALT [µkat/L]	0.22 (0.20–0.24)	0.29 (0.26–0.30)	0.25 (0.24–0.31)	**0.008**	C<H, P
Cholesterol [mmol/L]	4.32 (4.07–5.36)	4.37 (4.27–5.50)	4.61 (4.32–4.97)	0.699	
HDL cholesterol [mmol/L]	1.82 (1.74–2.09)	1.93 (1.65–2.10)	1.82 (1.52–2.07)	0.739	
LDL cholesterol [mmol/L]	2.39 (1.98–3.35)	2.55 (2.31–3.39)	2.74 (2.29–3.26)	0.643	
Triglycerides [mmol/L]	0.74 (0.67–0.81)	0.85 (0.61–1.30)	0.77 (0.70–0.97)	0.454	

Abbreviations in multiple comparison: C—control group; H—women with IH; P—women with PCOS; The ‘<’ symbol indicates that the analyte levels in the group on the left side are statistically significantly lower than the concentrations of the analyte in the groups on the right side of the symbol. Abbreviation of metabolic characteristics: ALP—alkaline phosphatase, ALT—alanine transaminase, AST—aspartate transaminase, GGT—gamma-glutamyl transferase, HOMA-IR—homeostatic model assessment for insulin resistance. p-values lower than 0.05 are marked in bold.

**Table 2:** Spearman correlations between 7-oxygenated metabolites of DHEA and the cytokine panel in all premenopausal women; n = 51: 20 controls, 12 women with IH, and 19 women with PCOS.

	7αOH-DHEA		7βOH-DHEA		7-ketoDHEA	
	r	*p*-Value	r	*p*-Value	r	*p*-Value
IL-1ra	**0.313**	**0.028**	**0.311**	**0.030**	**0.311**	**0.029**
IL-1β	**0.397**	**0.006**	**0.604**	**<0.0001**	**0.467**	**0.001**
IL-2	0.204	0.159	0.261	0.070	**0.298**	**0.037**
IL-4	0.214	0.139	0.239	0.099	0.224	0.122
IL-5	−0.012	0.938	0.179	0.258	0.144	0.364
IL-6	0.195	0.180	0.232	0.109	0.222	0.125
IL-7	0.142	0.330	0.214	0.140	0.244	0.091
IL-8	0.076	0.605	0.100	0.494	0.137	0.349
IL-9	0.176	0.227	0.229	0.114	0.216	0.136
IL-10	0.130	0.546	0.327	0.119	0.126	0.556
IL-12(p70)	0.248	0.104	**0.322**	**0.033**	**0.346**	**0.021**
IL-13	**0.298**	**0.038**	**0.378**	**0.007**	**0.366**	**0.010**
IL-15	0.101	0.509	0.261	0.083	0.232	0.125
IL-17	0.239	0.110	**0.367**	**0.012**	**0.294**	**0.047**
Eotaxin	−0.096	0.513	−0.030	0.840	−0.103	0.480
FGF basic	0.031	0.835	0.076	0.602	0.080	0.584
GM-CSF	0.179	0.219	0.221	0.128	0.233	0.107
G-CSF	0.203	0.162	0.250	0.083	0.202	0.165
IFN-γ	0.228	0.115	0.222	0.125	0.275	0.056
IP-10	0.095	0.518	0.192	0.185	0.102	0.484
MCP-1(MCAF)	0.250	0.083	0.236	0.103	0.241	0.095
MIP-1α	**0.292**	**0.042**	0.275	0.056	**0.305**	**0.033**
MIP-1β	0.111	0.446	**0.402**	**0.004**	0.176	0.226
PDGF-BB	−0.046	0.754	0.026	0.858	−0.013	0.932
RANTES	−0.135	0.356	0.075	0.611	−0.095	0.517
TNF-α	0.159	0.276	0.181	0.214	0.215	0.138
VEGF	0.040	0.786	0.106	0.474	0.103	0.485

Abbreviations of cytokines. IL—interleukin, FGF basic—fibroblast growth factor basic, G-CSF—granulocyte colony stimulating factor, GM-CSF—granulocyte macrophage colony stimulating factor, IFN-γ—interferon γ, IP-10—interferon γ-inducible protein, 10 kDa, MCAF/MCP-1—monocyte chemotactic and activating factor, MIP-1α—macrophage inflammatory protein 1α, PDGF-BB—platelet-derived growth factor, two B subunits, RANTES—regulated on activation, normal T expressed and secreted chemokine, TNF-α—tumor necrosis factor α, VEGF—vascular endothelial growth factor. p-values lower than 0.05 are marked in bold.

**Table 3:** Hormonal profile in the control group, the group of patients with idiopathic hirsutism, and the group of patients with PCOS.

	Controls (n = 20)	Women with IH (n = 12)	Women with PCOS (n = 10)	*p*-Value	Multiple Comparison
AMH [ng/mL]	2.52 (2.11–3.53)	2.14 (0.83–3.62)	8.53 (6.14–10.95)	**0.000**	C, H<P
SHBG [nmol/L]	68.9 (51.8–91.3)	57.3 (48.8–64.7)	44.3 (27.4–75.3)	0.131	
LH [IU/L]	6.6 (5.5–8.0)	7.1 (4.9–9.5)	9.3 (7.0–11.6)	0.082	
FSH [IU/L]	7.1 (5.5–9.0)	5.9 (5.1–7.5)	6.1 (3.3–7.3)	0.509	
LH/FSH	0.83 (0.69–1.13)	1.21 (0.85–1.55)	1.71 (1.53–2.21)	**<0.001**	C, H<P
Pregnenolone [ng/mL]	0.995 (0.577–1.274)	1.441 (0.901–2.670)	1.182 (0.856–2.295)	0.170	
17OHPreg [ng/mL]	5.495 (3.669–11.617)	5.565 (2.550–6.748)	9.105 (3.226–13.095)	0.282	
Progesterone [ng/mL]	0.123 (0.098–0.185)	0.098 (0.098–0.098)	0.098 (0.098–0.295)	0.400	
17OHProg [ng/mL]	0.433 (0.367–0.517)	0.503 (0.340- 0.695)	0.839 (0.560–0.882)	**0.011**	C, H<P
11DOF [ng/mL]	0.282 (0185–0.611)	0.310 (0.185–0.540)	0.630 (0.211–0.884)	0.451	
11DOC [ng/mL]	0.022 (0.014–0.042)	0.014 (0.014–0.031)	0.030 (0.014–0.075)	0.547	
Cortisol [ng/mL]	188 (138–231)	197 (165–225)	177 (132–286)	0.727	
Cortisone [ng/mL]	42.74 (40.05–47.16)	44.58 (37.56–51.81)	46.52 (39.50–49.73)	0.989	
Corticosterone [ng/mL]	2.099 (1.237–4.38)	2.452 (1.544–3.029)	3.289 (1.237–6.82)	0.574	
Aldosterone [ng/mL]	0.134 (0.068–0.179)	0.092 (0.055–0.148)	0.126 (0.108–0.255)	0.758	
DHEA [ng/mL]	7.64 (4.63–11.53)	6.28 (5.05–8.33)	12.62 (7.85–16.12)	**0.041**	H<P
T [ng/mL]	0.235 (0.202–0.269)	0.229 (0.096–0.301)	0.362 (0.264–0.460)	**0.015**	C, H<P
Free T [pmol/L]	25.2 (21.4–35.9)	32.3 (26.1–46.7)	46.8 (36.8–59.4)	**0.003**	C<P
Epitestosterone [ng/mL]	0.005 (0.004–0.010)	0.007 (0.003–0.010)	0.014 (0.009–0.018)	**0.003**	C, H<P
A4 [ng/mL]	1.195 (0.945–1.431)	1.135 (0.643–1.638)	2.119 (1.706–2.625)	**0.001**	C, H<P
DHT [ng/mL]	0.098 (0.066–0.118)	0.066 (0.066–0.076)	0.102 (0.086–0.133)	**0.045**	H<C, P
11βOHA4 [ng/mL]	0.701 (0.414–0.908)	0.458 (0.325–0.631)	1.029 (0.527–1.404)	**0.036**	H<P
11KT [ng/mL]	0.407 (0.329–0.503)	0.303 (0.191–0.396)	0.483 (0.325–0.647)	**0.022**	H<C, P
11OHT [ng/mL]	0.138 (0.100–0.148)	0.085 (0.060–0.113)	0.145 (0.088–0.226)	**0.027**	H<C, P
7αOHDHEA [ng/mL]	0.358 (0.228–0.596)	0.285 (0.223–0.326)	0.565 (0.328–0.632)	**0.017**	H<C, P
7βOHDHEA [ng/mL]	0.148 (0.066–0.200)	0.098 (0.081–0.136)	0.201 (0.158–0.257)	**0.031**	H<P
7KDHEA [ng/mL]	0.083 (0.065–0.110)	0.062 (0.045–0.078)	0.102 (0.079–0.116)	**0.005**	H<C, P
Estrone [ng/mL]	0.033 (0.026–0.037)	0.028 (0.022–0.052)	0.047 (0.035–0.057)	**0.031**	C, H<P
17β-Estradiol [ng/mL]	0.021 (0.016–0.030)	0.027 (0.015–0.037)	0.023 (0.021–0.030)	0.818	

Abbreviations in multiple comparisons: C—controls, H—women with IH, P—women with PCOS. Abbreviations of hormones: AMH—Anti-Müllerian hormone; SHBG—sex hormone binding globulin; FSH—follicle-stimulating hormone; LH—luteinizing hormone; 17OHPreg—17-hydroxypregnenolone; 11DOC—11-deoxycorticosterone; 17OHProg—17-hydroxyprogesterone; 11DOF—11-deoxycortisol; 21DOF—21-deoxycortisol; DHEA—dehydroepiandrosterone; T—testosterone; A4—androstenedione; DHT—dihydrotestosterone; 11βOHA4—11β-hydroxyandrostenedione; 11KT—11-ketotestosterone; 11OHT—11β-hydroxytestosterone; 7αOHDHEA—7α-hydroxydehydroepiandrosterone; 7βOHDHEA—7β-hydroxydehydroepiandrosterone; 7KDHEA—7-ketodehydroepiandrosterone. p-values lower than 0.05 are marked in bold.

**Table 4:** Spearman’s correlations between classic and 11-oxygenated androgens and metabolic parameters (n = 51: 20 controls, 12 women with IH, and 19 women with PCOS).

	11βOHA4		11KT		11OHT		DHEA		A4		T		DHT	
	r	*p*-Value	r	*p*-Value	r	*p*-Value	r	*p*-Value	r	*p*-Value	r	*p*-Value	r	*p*-Value
Age	−0.100	0.485	−0.145	0.309	0.127	0.374	−0.137	0.338	**−0.329**	**0.018**	−0.101	0.481	−0.087	0.542
BMI	−0.100	0.504	−0.002	0.990	0.052	0.727	0.083	0.580	0.132	0.377	0.277	0.059	−0.226	0.128
HOMA-IR	0.244	0.098	**0.366**	**0.011**	0.233	0.115	0.257	0.081	**0.471**	**0.001**	**0.357**	**0.014**	−0.094	0.530
Insulin	0.242	0.091	**0.375**	**0.007**	0.236	0.100	0.196	0.173	**0.436**	**0.002**	**0.347**	**0.014**	−0.094	0.517
Cholesterol	−0.165	0.253	−0.225	0.117	−0.056	0.700	−0.203	0.158	−0.150	0.299	−0.078	0.589	−0.320	0.024
Triglycerides	0.034	0.817	0.137	0.345	0.061	0.676	0.093	0.521	0.220	0.125	0.246	0.085	−0.149	0.303
LDL cholesterol	−0.136	0.345	−0.110	0.449	−0.001	0.997	−0.128	0.377	−0.105	0.466	−0.083	0.568	**−0.286**	**0.044**
HDL Cholesterol	−0.077	0.596	−0.171	0.234	−0.222	0.121	−0.249	0.081	−0.128	0.374	−0.048	0.743	−0.077	0.595
Hemoglobin A1c	0.072	0.621	0.066	0.654	0.128	0.382	0.099	0.498	0.258	0.073	0.187	0.198	−0.228	0.115
Glucose	0.104	0.474	0.068	0.637	0.119	0.410	0.271	0.057	0.329	0.020	**0.334**	**0.018**	0.037	0.798
ALP	0.135	0.351	0.276	0.052	0.177	0.218	0.032	0.826	0.226	0.114	0.126	0.382	−0.132	0.362
ALT	0.040	0.784	−0.030	0.838	0.038	0.791	0.136	0.346	**0.358**	**0.011**	**0.437**	**0.002**	−0.170	0.237
AST	0.007	0.964	0.026	0.859	0.054	0.708	0.015	0.915	0.270	0.058	**0.353**	**0.012**	−0.133	0.356
GGT	0.143	0.323	0.263	0.066	0.241	0.092	0.252	0.077	0.369	0.008	**0.373**	**0.008**	−0.030	0.834

Abbreviation of analytes: ALP—alkaline phosphatase, ALT—alanine transaminase, AST—aspartate transaminase, GGT—gamma-glutamyl transferase, HOMA-IR—homeostatic model assessment for insulin resistance, 11βOHA4—11β-hydroxyandrostenedione, 11KT—11-ketotestosterone, 11OHT—11β-hydroxytestosterone, DHEA—dehydroepiandrosterone, A4—androstenedione, T—testosterone, DHT—dihydrotestosterone. p-values lower than 0.05 are marked in bold.

**Table 5:** ED levels and their occurrences in control group, group of patients with idiopathic hirsutism (IH), and group of patients with PCOS and the multiple comparisons.

	Controls (n = 20)	Women with IH (n = 12)	Women with PCOS (n = 10)	*p*-Value
∑parabens	0.013 (0.013–0.081)	0.0395 (0.013–0.753)	0.013 (0.013–0.087)	0.448
MP [ng/mL]	0.013 (0.013–0.070)	0.0395 (0.013–0.583)	0.013 (0.013–0.072)	0.405
MP occurrence (%)	35	58	40	
BPA [ng/mL]	0.123 (0.041–0.289)	0.107 (0.092–0.162)	0.053 (0.014–0.113)	0.234
BPA occurrence (%)	85	92	60	
BPS [ng/mL]	0.028 (0.011–0.039)	0.011 (0.011–0.039)	0.035 (0.028–0.051)	0.465
BPS occurrence (%)	65	50	90	
oxybenzone [ng/mL]	0.014 (0.014–0.053)	0.014 (0.014–0.054)	0.014 (0.014–0.039)	0.938
oxybenzone occurrence (%)	35	33	30	

Abbreviations: MP—methylparaben, BPA—bisphenol A, BPS—bisphenol S.

**Table 6:** Comparisons of BMI, AMH, EDs with the highest occurrences, and classic and 11-oxygenated androgens between normal weight and obese women with PCOS.

	Normal Weight Women with PCOS (n = 10)	Obese Women with PCOS (n = 9)	*p*-Value
BMI [kg/m^2^]	21.6 (18.4–24.5)	34.6 (32.9–35.7)	**<0.0001**
AMH [ng/mL]	8.525 (6.140–10.950)	5.500 (4.535–6.555)	**0.025**
BPA [ng/mL]	0.053 (0.014–0.113)	0.193 (0.138–0.302)	**0.014**
BPS [ng/mL]	0.035 (0.027–0.055)	0.029 (0.011–0.041)	0.206
MP [ng/mL]	0.013 (0.013–0.072)	0.013 (0.013–0.070)	1.000
oxybenzone [ng/mL]	0.014 (0.014–0.039)	0.014 (0.014–0.078)	0.767
11βOHA4 [ng/mL]	1.029 (0.527–1.404)	0.618 (0.377–0.958)	0.149
11KT [ng/mL]	0.483 (0.325–0.647)	0.436 (0.267–0.637)	0.545
11OHT [ng/mL]	0.145 (0.088–0.226)	0.114 (0.095–0.170)	0.589
A4 [ng/mL]	2.119 (1.706–2.625)	1.547 (1.130–2.310)	0.128
T [ng/mL]	0.362 (0.264–0.460)	0.315 (0.215–0.462)	0.441
DHEA [ng/mL]	12.625 (7.850–16.119)	7.095 (6.133–12.213)	0.330
DHT [ng/mL]	0.102 (0.086–0.133)	0.064 (0.059–0.103)	0.084

Abbreviations of analytes: AMH—Anti-Müllerian hormone; BPA—bisphenol A; BPS—bisphenol S; MP—methylparaben; 11βOHA4—11β-hydroxyandrostenedione; 11KT—11-ketotestosterone; 11OHT—11β-hydroxytestosterone; A4—androstenedione; T—testosterone; DHEA—dehydroepiandrosterone; DHT—dihydrotestosterone. *p*-values lower than 0.05 are marked in bold.

## Data Availability

Data will be made available on request.

## Supplementary Materials

The following supporting information can be downloaded at: https://www.mdpi.com/article/10.3390/ijms25179691/s1.
