# Supplementary material for: The Role of 11-Oxygenated Androgens and Endocrine Disruptors in Androgen Excess Disorders in Women
Source: Int J Mol Sci. 2024 Sep 7;25(17):9691. doi: 10.3390/ijms25179691 (PMC11395667; doi:10.3390/ijms25179691)
Supplement: Supplementary file 1 [file ijms-25-09691-s001.zip › ijms-3151684-SI.pdf]

Supplementary Material

The Role of 11-Oxygenated Androgens and Endocrine Disruptors in Androgen Excess Disorders in Women

Jana Vitku[1] *, Anezka Varausova[1], Tereza Skodova[1], Lucie Kolatorova[1], Michala Vosatkova[2], Josef Vcelak[3], Jana Vrbikova[4], Marketa Simkova[1] and Michaela Svojtkova[1]

[1] Department of Steroids and Proteofactors, Institute of Endocrinology, Narodni 8, 116 94 Prague, Czech Republic
[2] Department of Clinical Biochemistry, Institute of Endocrinology, Narodni 8, 116 94 Prague, Czech Republic
[3] Department of Molecular Endocrinology, Institute of Endocrinology, Narodni 8, 116 94 Prague, Czech Republic
[4] Department of Clinical Endocrinology, Institute of Endocrinology, Narodni 8, 116 94 Prague, Czech Republic
* Correspondence: jvitku@endo.cz; Tel.:+420-224-905-238

Table of Contents

Table S1. Comparison of cytokine levels (pg/mL) in control group, group of women with idiopathic hirsutism (IH) and group of women with PCOS……...……………………………....2

Table S2. Comparison of conjugated steroid levels in control group, group of women with idiopathic hirsutism (IH) and group of women with PCOS ……………………...………...….3

Table S3. Spearman's correlations between EDs with the highest occurrence (BPA, BPS, MP and oxybenzone) and metabolic parameters……………………………………………………4

Table S4. Spearman's correlations between the EDs and cytokine panel together with immunomodulatory 7-oxygenated metabolites od DHEA…………………...………………..5

Table S5. Spearman's correlations between EDs with the highest occurrence (BPA, BPS, MP and oxybenzone) and classic as well as 11-oxygenated androgens………………...…………6

Table S6. Comparison of cytokine levels (pg/mL) in normal weight and obese women with PCOS……………………………………………………………………………………………7

Table S1. Comparison of cytokine levels (pg/mL) in control group, group of women with idiopathic hirsutism (IH) and group of women with PCOS

Cytokines [pg/mL]	Controls (n=20)	Women with IH (n=12)	Women with PCOS (n=10)	p-value	Multiple comparison
IL-1ra	570 (399-720)	454 (328-533)	395 (311-533)	0.099	
IL-1β	1.57 (1.42-1.88)	1.31 (1.24-1.46)	1.49 (1.33-1.75)	0.082	
IL-2	18.9 (9.27-25.53)	14.08 (10.62-23.38)	15.52 (10.35-25.91)	0.972	
IL-4	2.93 (2.06-3.42)	2.47 (2.01-3.06)	2.03 (1.75-2.72)	0.338	
IL-5	50.27 (27.68-87.34)	28.42 (6.00-42.41)	45.62 (29.39-249.55)	0.121	
IL-6	24.24 (10.51-45.66)	13.60 (8.41-56.77)	82.40 (7.67-206.21)	0.158	
IL-7	17.9 (12.65-21.29)	13.75 (11.43-16.83)	12.25 (10.2-14.05)	0.075	
IL-8	22.59 (13.59-25.13)	17.41 (11.67-21.43)	14.65 (10.82-16.55)	0.178	
IL-9	50.15 (45.93-53.62)	47.93 (44.97-49.41)	45.40 (44.55-47.19)	0.108	
IL-10	7.56 (4.97-9.37)	5.88 (4.43-7.62)	5.65 (4.22-6.71)	0.131	
IL-12(p70)	19.02 (10.84-21.12)	11.93 (8.81 (16.71)	18.24 (7.28-28.47)	0.667	
IL-13	8.13 (5.42-9.88)	6.45 (4.85-8.43)	5.82 (4.85-7.07)	0.198	
IL-15	569 (290-692)	428 (299-570)	920 (387-1093)	0.320	
IL-17	19.04 (14.26-23.69)	16.47 (11.65-18.65)	15.04 (11.92-16.46)	0.179	
Eotaxin	59.60 (50.24-73.76)	75.83 (68.23-83.83)	74.17 (59.16-80.35)	0.067	
FGF basic	56.41 (42.07-64.85)	55.22 (47.8-62.98)	50.74 (43.42-61.47)	0.948	
GM-CSF	15.91 (8.96-22.40)	10.89 (8.65-19.84)	21.16 (8.87-44.67)	0.317	
G-CSF	236 (153-315)	187 (136-215)	179 (124-247)	0.111	
IFN-γ	19.86 (12.12-35.78)	15.35 (10.91-25.7)	15.99 (11.04-24.70)	0.669	
IP-10	699 (600-871)	723 (583-802)	751 (522-841)	0.868	
MCP-1(MCAF)	20.39 (17.72-25.06)	22.11 (20.68-22.95)	22.74 (15.39-26.74)	0.808	
MIP-1α	2.77 (2.44-3.34)	2.63 (2.05-3.15)	2.15 (1-96-3.00)	0.224	
MIP-1β	906 (860-956)	943 (876-976)	921 (883-969)	0.490	
PDGF-BB	200 (130-288)	232 (196-398)	331 (277-432)	**0.009**	C<P
RANTES	4733 (3795-5582)	5324 (4855 -6700)	5680 (4737-6426)	0.113	
TNF-α	72.62 (56.74-85.98)	60.55 (56.10-67.48)	60.54 (45.43-69.97)	0.237	
VEGF	460 (177-868)	333 (281-1206)	1029 (487-1952)	0.191	

Abbreviations in multiple comparison: C – control group; P – women with PCOS

Abbreviations of cytokines: IL – interleukin, FGF basic - fibroblast growth factor basic, G-CSF - granulocyte colony stimulating factor, GM-CSF - granulocyte macrophage colony stimulating factor, IFN-γ - interferon γ, IP-10- interferon γ-inducible protein, 10 kDa, MCAF/MCP-1 - monocyte chemotactic and activating factor, MIP-1α - macrophage inflammatory protein 1α, PDGF-BB - platelet-derived growth factor, two B subunits, RANTES - regulated on activation, normal T expressed and secreted chemokine, TNF-α - tumor necrosis factor α, VEGF - vascular endothelial growth factor

Table S2. Comparison of conjugated steroids' levels [ng/mL] in control group, group of women with idiopathic hirsutism (IH) and group of women with PCOS

**Conjugated steroids [ng/mL]**	**Controls (n=20)**	**Women with IH (n=12)**	**Women with PCOS (n=10)**	**p-value**	**Multiple comparison**
Pregnenolone	33.33 (21.08-42.21)	31.72 (27.00-42.38)	35.07 (30.58-48.09)	0.772	
17OHPreg	0.907 (0.383-1.429)	1.694 (0.614-3.770)	1.248 (0.295 -2.681)	0.347	
17OHProg	0.970 (0.676-1.439)	0.960 (0.672-1.193)	0.940 (0.774-1.356)	0.806	
11DOC	0.014 (0.014-0.014)	0.014 (0.014-0.014)	0.023 (0.014-0.027)	0.054	C,H<P
21DOF	0.315 (0.276-0.418)	0.327 (0.264-0.365)	0.295 (0.252-0.369)	0.624	
Corticosterone	1.153 (0.944-1.541)	1.093 (0.807-1.512)	1.401 (1.056-1.421)	0.559	
DHEA	551 (491-651)	457 (371-591)	626 (334-720)	0.198	
T	0.096 (0.096-0.133)	0.096 (0.096-0.190)	0.096 (0.096-0.096)	0.380	
Epitestosterone	0.150 (0.094-0.218)	0.137 (0.098-0.184)	0.170 (0.164-0.270)	0.279	
A4	0.629 (0.500-0.722)	0.461 (0.360-0.682)	0.658 (0.534-0.857)	0.051	
11KT	0.024 (0.014-0.042)	0.037 (0.024-0.058)	0.046 (0.022-0.085)	0.123	
7αOHDHEA	0.173 (0.069-0.530)	0.220 (0.069-0.365)	0.337 (0.134-1.335)	0.394	
7βOHDHEA	0.075 (0.036-0.121)	0.058 (0.036-0.222)	0.160 (0.060-0.449)	0.145	
7KDHEA	0.166 (0.068-0.221)	0.065 (0.053 -0.111)	0.219 (0.074-0.488)	**0.009**	H<P
Estrone	0.256 (0.203-0.322)	0.216 (0.161-0.342)	0.258 (0.215-0.320)	0.688	
Estriol	0.166 (0.145-0.232)	0.200 (0.164-0.244)	0.138 (0.008-0.216)	0.137	

Abbreviations in multiple comparison: C – control group; H – women with IH; P – women with PCOS

Abbreviations of steroids: 17OHPreg – 17-hydroxypregnenolone; 17OHProg - 17-hydroxyprogesterone; 11DOC - 11-deoxycorticosterone; 21DOF - 21-deoxycortisol; DHEA – dehydroepiandrosterone; T – testosterone; A4 – androstenedione; 11KT – 11-ketotestosterone; 7αOHDHEA - 7α-hydroxydehydroepiandrosterone; 7βOHDHEA- 7β-hydroxydehydroepiandrosterone; 7KDHEA - 7-ketodehydroepiandrosterone

Table S3 Spearman's correlations between EDs with the highest occurrence (BPA, BPS, MP and oxybenzone) and metabolic parameters

	BPA		BPS		MP		Oxybenzone	
	**r**	**p-value**	**r**	**p-value**	**r**	**p-value**	**r**	**p-value**
Age	0.071	0.622	-0.020	0.888	0.121	0.397	-0.125	0.384
BMI	0.283	0.056	-0.204	0.169	-0.039	0.794	0.061	0.686
HOMA-IR	0.061	0.687	-0.055	0.712	-0.085	0.570	0.000	1.000
Insulin	0.055	0.707	-0.083	0.567	-0.116	0.423	0.024	0.870
Cholesterol	0.058	0.693	-0.032	0.823	-0.180	0.210	0.059	0.686
Triglycerides	0.085	0.563	0.048	0.741	0.042	0.773	-0.020	0.892
LDL cholesterol	0.098	0.503	-0.074	0.609	-0.248	0.082	0.165	0.253
HDL Cholesterol	-0.206	0.156	0.082	0.573	0.084	0.560	-0.127	0.381
Hemoglobin A1c	0.011	0.939	-0.001	0.996	-0.009	0.950	0.085	0.563
Glucose	-0.115	0.433	-0.125	0.387	-0.042	0.774	-0.053	0.713
ALP	0.147	0.314	0.034	0.817	-0.039	0.790	0.208	0.148
ALT	0.139	0.342	**-0.323**	**0.022**	0.024	0.867	-0.053	0.716
AST	-0.113	0.440	-0.167	0.247	-0.193	0.180	-0.025	0.862
GGT	0.277	0.054	0.263	0.065	0.006	0.964	0.101	0.487

Table S4. Spearman's correlations between the EDs and cytokine panel together with immunomodulatory 7-oxygenated metabolites od DHEA

	BPA		BPS		MP		Oxybenzone	
	**r**	**p-value**	**r**	**p-value**	**r**	**p-value**	**r**	**p-value**
7αOHDHEA	-0.038	0.795	**0.292**	**0.037**	0.026	0.857	0.077	0.593
7βOHDHEA	0.013	0.927	0.237	0.094	0.013	0.929	0.008	0.957
7KDHEA	0.042	0.774	**0.443**	**0.001**	0.078	0.587	0.075	0.602
IL-1ra	-0.026	0.861	0.129	0.376	-0.045	0.759	-0.016	0.911
IL-1β	0.027	0.858	0.216	0.146	-0.059	0.694	0.010	0.947
IL-2	0.006	0.969	0.150	0.302	-0.083	0.571	0.065	0.656
IL-4	0.029	0.841	0.173	0.233	0.013	0.929	-0.010	0.945
IL-5	-0.126	0.426	-0.145	0.360	-0.109	0.494	-0.079	0.617
IL-6	-0.058	0.691	0.101	0.492	0.026	0.859	-0.013	0.928
IL-7	0.091	0.535	0.113	0.438	-0.017	0.905	0.038	0.797
IL-8	0.071	0.630	0.134	0.359	0.045	0.758	-0.064	0.660
IL-9	-0.013	0.931	0.174	0.233	-0.052	0.724	0.078	0.596
IL-10	0.091	0.673	0.290	0.170	0.187	0.381	-0.112	0.604
IL-12(p70)	0.060	0.699	0.125	0.420	-0.024	0.878	0.042	0.786
IL-13	0.141	0.334	0.142	0.331	0.069	0.637	0.017	0.910
IL-15	0.004	0.980	0.108	0.481	-0.097	0.526	-0.015	0.922
IL-17	0.019	0.903	0.065	0.666	-0.062	0.683	-0.084	0.579
Eotaxin	0.012	0.934	0.029	0.845	0.060	0.681	-0.151	0.301
FGF basic	-0.098	0.505	0.161	0.270	-0.010	0.947	-0.069	0.638
GM-CSF	-0.003	0.985	-0.007	0.960	-0.028	0.848	0.022	0.880
G-CSF	0.089	0.545	0.108	0.460	-0.115	0.431	-0.037	0.803
IFN-γ	0.057	0.700	0.041	0.780	-0.124	0.396	0.076	0.606
IP-10	-0.036	0.805	**-0.377**	**0.008**	-0.118	0.419	0.030	0.837
MCP-1(MCAF)	-0.090	0.540	0.113	0.438	-0.134	0.358	0.047	0.749
MIP-1α	0.015	0.921	0.111	0.446	0.062	0.674	-0.088	0.549
MIP-1β	0.061	0.676	-0.059	0.686	-0.037	0.800	0.127	0.383
PDGF-BB	-0.095	0.514	0.024	0.872	0.021	0.885	-0.021	0.887
RANTES	0.105	0.473	-0.067	0.647	-0.013	0.928	-0.007	0.961
TNF-α	-0.087	0.551	0.077	0.597	-0.139	0.341	-0.201	0.165
VEGF	0.022	0.881	-0.024	0.870	-0.035	0.815	0.081	0.586

Abbreviations of analytes. 7αOHDHEA - 7α-hydroxydehydroepiandrosterone, 7βOHDHEA - 7β-hydroxydehydroepiandrosterone, 7KDHEA – 7-ketodehydroepiandrosterone, IL – interleukin, FGF basic - fibroblast growth factor basic, G-CSF - granulocyte colony stimulating factor, GM-CSF - granulocyte macrophage colony stimulating factor, IFN-γ - interferon γ, IP-10- interferon γ-inducible protein, 10 kDa, MCAF/MCP-1 - monocyte chemotactic and activating factor, MIP-1α - macrophage inflammatory protein 1α, PDGF-BB - platelet-derived growth factor, two B subunits, RANTES - regulated on activation, normal T expressed and secreted chemokine, TNF-α - tumor necrosis factor α, VEGF - vascular endothelial growth factor

Table S5. Spearman's correlations between EDs with the highest occurrence (BPA, BPS, MP and oxybenzone) and classic as well as 11-oxygenated androgens

	**BPA**		**BPS**		**MP**		**Oxybenzone**	
	**r**	**p-value**	**r**	**p-value**	**r**	**p-value**	**r**	**p-value**
11βOHA4	-0.231	0.107	0.137	0.336	0.031	0.827	-0.034	0.813
11KT	-0.153	0.288	0.213	0.133	-0.126	0.38	0.083	0.562
11OHT	-0.097	0.501	0.175	0.221	-0.06	0.676	0.143	0.316
DHEA	-0.153	0.289	0.176	0.216	0.077	0.589	0.012	0.934
A4	**-0.288**	**0.043**	0.137	0.339	-0.131	0.360	-0.040	0.779
T	**-0.289**	**0.042**	0.123	0.390	-0.035	0.807	-0.065	0.649
DHT	-0.142	0.324	0.256	0.070	0.138	0.333	-0.065	0.652

Abbreviations: 11βOHA4 - 11β-hydroxyandrostenedione; 11KT – 11-ketotestosterone; 11OHT – 11β-hydroxytestosterone; DHEA – dehydroepiandrosterone; A4 – androstenedione; T – testosterone; DHT – dihydrotestosterone

Table S6. Comparison of cytokine levels (pg/mL) in normal weight and obese women with PCOS

Cytokines [pg/mL]	normal weight women with PCOS (n=10)	obese women with PCOS (n=9)	p-value
IL-1ra	394.8 (310.7-533.0)	632.4 (461.0-723.2)	**0.022**
IL-1β	1.485 (1.33-1.75)	1.650 (1.505-1.760)	0.269
IL-2	15.52 (10.35-25.91)	21.34 (19.61-25.36)	0.225
IL-4	2.03 (1.75-2.72)	3.19 (2.22-3.36)	0.198
IL-5	45.62 (29.39-249.55)	105.5 (75.46-245.13)	0.400
IL-6	82.4 (7.67-206.21)	79.66 (41.59-155.14)	0.588
IL-7	12.25 (10.20-14.05)	17.41 (15.62-20.88)	**0.005**
IL-8	14.65 (10.82-16.55)	20.16 (15.65-23.28)	0.119
IL-9	45.4 (44.55-47.19)	49.62 (46.69-54.67)	0.085
IL-10	5.65 (4.22-6.71)	8.08 (5.02-8.67)	0.198
IL-12(p70)	18.24 (7.28-28.47)	23.36 (16.97-35.73)	0.356
IL-13	5.82 (4.85-7.07)	10.02 (7.23-10.87)	**0.003**
IL-15	919.6 (386.9-1092.8)	938.7 (741.3-1107.8)	0.905
IL-17	15.04 (11.92-16.46)	21.21 (14.85-23.69)	0.079
Eotaxin	74.17 (59.16-80.35)	53.04 (51.22-61.02)	0.014
FGF basic	50.74(43.42-61.47)	64.48 (53.92-67.42)	0.121
GM-CSF	21.16 (8.87-44.67)	29.52 (26.02-57.36)	0.255
G-CSF	179.1 (123.8-247.2)	269.5 (169.0-284.3)	0.113
IFN-γ	15.99 (11.04-24.70)	31.64 (22.48-40.95)	**0.028**
IP-10	750.8 (522.1-841.4)	675.3 (566.4-814.6)	0.549
MCP-1(MCAF)	22.74 (15.38-26.74)	21.25 (18.89-23.11)	0.423
MIP-1α	2.15 (1.96-3.00)	3.15 (2.59-3.40)	0.054
MIP-1β	920.5 (883.2-968.7)	955.3 (850.5-1009.5)	0.871
PDGF-BB	331.0 (277.0-432.9)	282.1 (216.0-345.0)	0.557
RANTES	5680 (4737-6426)	4402 (4184-5670)	0.384
TNF-α	60.54 (45.43-69.97)	82.32 (63.84-90.53)	0.165
VEGF	1028.8 (487.3-1952.5)	1089 (805.1-1843.4)	0.459

Abbreviations of cytokines. IL – interleukin, FGF basic - fibroblast growth factor basic, G-CSF - granulocyte colony stimulating factor, GM-CSF - granulocyte macrophage colony stimulating factor, IFN-γ - interferon γ, IP-10- interferon γ-inducible protein, 10 kDa, MCAF/MCP-1 - monocyte chemotactic and activating factor, MIP-1α - macrophage inflammatory protein 1α, PDGF-BB - platelet-derived growth factor, two B subunits, RANTES - regulated on activation, normal T expressed and secreted chemokine, TNF-α - tumor necrosis factor α, VEGF - vascular endothelial growth factor
